# Supplementary material for: Sex differentiation, divergence, and introgression in the leishmaniasis vector Lutzomyia longipalpis
Source: bioRxiv. 2025 Nov 4:2025.04.08.647763. Preprint. [Version 2] doi: 10.1101/2025.04.08.647763 (PMC12637706; doi:10.1101/2025.04.08.647763)
Supplement: Supplement 1 [file media-1.pdf]

SUPPLEMENTARY TABLES

Table S1. Sample information for short read genomic data used in this study. This includes the collections from Colombia and a list of accession numbers of the previously published reads used in this study.

Run	Site	Sex
LU_LONG_M_131_CAATTAAC-CGAGATAT_S34	Ricaurte, Colombia	Male
JB_LM30-lutzomyia_male_42_021921_TCCTGAGC-AAGGCTAT_S79	Ricaurte, Colombia	Male
JB_LM11-lutzomyia_male_23_021921_CGTACTAG-CCTAGAGT_S58	Ricaurte, Colombia	Male
JB_LF33-lutzomyia_female_100_021921_CGAGGCTG-GAGCCTTA_S26	Ricaurte, Colombia	Female
JB_LF13-lutzomyia_female_80_021921_TAGGCATG-CCTAGAGT_S4	Ricaurte, Colombia	Female
JB_LF4-lutzomyia_female_71_021921_GGACTCCT-TTCTAGCT_S44	Ricaurte, Colombia	Female
JB_LM3-lutzomyia_male_15_021921_TAAGGCGA-CCTAGAGT_S80	Ricaurte, Colombia	Male
LU_LONG_F_P12_GGACTTGG-CGCAGACG_S20	Ricaurte, Colombia	Female
JB_LM1-lutzomyia_male_13_021921_TAAGGCGA-TCGACTAG_S67	Ricaurte, Colombia	Male
JB_LF52-lutzomyia_female_119_021921_GCTCATGA-TTCTAGCT_S47	Ricaurte, Colombia	Female
JB_LM24-lutzomyia_male_36_021921_AGGCAGAA-TTATGCGA_S72	Ricaurte, Colombia	Male
JB_LM22-lutzomyia_male_34_021921_AGGCAGAA-AAGGCTAT_S70	Ricaurte, Colombia	Male
JB_LM19-lutzomyia_male_31_021921_AGGCAGAA-CCTAGAGT_S66	Ricaurte, Colombia	Male
JB_LM5-lutzomyia_male_17_021921_TAAGGCGA-CTATTAAG_S82	Ricaurte, Colombia	Male
JB_LF45-lutzomyia_female_112_021921_GTAGAGGA-CCTAGAGT_S39	Ricaurte, Colombia	Female
JB_LF51-lutzomyia_female_118_021921_GCTCATGA-TCGACTAG_S46	Ricaurte, Colombia	Female

JB_LM18-lutzomyia_male_30_021921_AGGCAGAA-TTCTAGCT_S65	Ricaurte, Colombia	Male
JB_LM2-lutzomyia_male_14_021921_TAAGGCGA-TTCTAGCT_S78	Ricaurte, Colombia	Male
LU_LONG_F_138_CATAGAGT-TGCCACCA_S5	Ricaurte, Colombia	Female
JB_LF48-lutzomyia_female_115_021921_GTAGAGGA-AAGGCTAT_S42	Ricaurte, Colombia	Female
LU_LONG_F_147_CTCACCAA-CTAGGCAA_S14	Ricaurte, Colombia	Female
JB_LF22-lutzomyia_female_89_021921_CTCTCTAC-GCGTAAGA_S14	Ricaurte, Colombia	Female
JB_LM7-lutzomyia_male_19_021921_TAAGGCGA-GAGCCTTA_S84	Ricaurte, Colombia	Male
LU_LONG_M_127_GCCACAGG-CATGCCAT_S30	Ricaurte, Colombia	Male
JB_LF7-lutzomyia_female_74_021921_GGACTCCT-CTATTAAG_S54	Ricaurte, Colombia	Female
LU_LONG_F_P19_AGTTCAGG-CCAACAGA_S26	Ricaurte, Colombia	Female
JB_LF18-lutzomyia_female_85_021921_TAGGCATG-TTATGCGA_S9	Ricaurte, Colombia	Female
LU_LONG_F_154_GTATGTTC-AACAGGAA_S18	Ricaurte, Colombia	Female
LU_LONG_M_128_ATTGTGAA-TGCATTGC_S31	Ricaurte, Colombia	Male
LU_LONG_F_135_GACGTCTT-GGTTCACC_S3	Ricaurte, Colombia	Female
JB_LM10-lutzomyia_male_22_021921_CGTACTAG-TTCTAGCT_S57	Ricaurte, Colombia	Male
LU_LONG_F_150_CGCTATGT-TCCGACAC_S17	Ricaurte, Colombia	Female
LU_LONG_F_P11_TTATAACC-GATATCGA_S19	Ricaurte, Colombia	Female
JB_LF11-lutzomyia_female_78_021921_TAGGCATG-TCGACTAG_S2	Ricaurte, Colombia	Female
LU_LONG_F_146_GAACCGCG-TGACCTTA_S13	Ricaurte, Colombia	Female

JB_LF40-lutzomyia_female_107_021921_AAGAGGCA-AAGGCTAT_S34	Ricaurte, Colombia	Female
LU_LONG_F_P18_TGGATCGA-GTGCGATA_S25	Ricaurte, Colombia	Female
LU_LONG_F_137_TGCGAGAC-CATTGTTG_S4	Ricaurte, Colombia	Female
JB_LF25-lutzomyia_female_92_021921_CTCTCTAC-GAGCCTTA_S17	Ricaurte, Colombia	Female
LU_LONG_F_143_CTGCTTCC-GATAGATC_S10	Ricaurte, Colombia	Female
JB_LF47-lutzomyia_female_114_021921_GTAGAGGA-CTATTAAG_S41	Ricaurte, Colombia	Female
JB_LM17-lutzomyia_male_29_021921_AGGCAGAA-TCGACTAG_S64	Ricaurte, Colombia	Male
LU_LONG_F_P16_GCTTGTCA-GAACATAC_S23	Ricaurte, Colombia	Female
JB_LF43-lutzomyia_female_110_021921_GTAGAGGA-TCGACTAG_S37	Ricaurte, Colombia	Female
LU_LONG_F_149_TATCGCAC-CTTAGTGT_S16	Ricaurte, Colombia	Female
JB_LF5-lutzomyia_female_72_021921_GGACTCCT-CCTAGAGT_S52	Ricaurte, Colombia	Female
LU_LONG_F_144_TCATCCTT-AGCGAGCT_S11	Ricaurte, Colombia	Female
JB_LM25-lutzomyia_male_37_021921_TCCTGAGC-TCGACTAG_S73	Ricaurte, Colombia	Male
LU_LONG_F_142_GGTCACGA-GTATTATG_S9	Ricaurte, Colombia	Female
LU_LONG_F_145_AGGTTATA-CAGTTCCG_S12	Ricaurte, Colombia	Female
LU_LONG_F_P20_GACCTGAA-TTGGTGAG_S27	Ricaurte, Colombia	Female
JB_LM16-lutzomyia_male_28_021921_CGTACTAG-TTATGCGA_S63	Ricaurte, Colombia	Male
JB_LM14-lutzomyia_male_26_021921_CGTACTAG-AAGGCTAT_S61	Ricaurte, Colombia	Male
LU_LONG_F_140_GTGAATAT-TCTCATTC_S7	Ricaurte, Colombia	Female

LU_LONG_F_148_TCTGTTGG-TCGAATGG_S15	Ricaurte, Colombia	Female
LU_LONG_F_141_AACTGTAG-ACGCCGCA_S8	Ricaurte, Colombia	Female
LU_LONG_F_P14_AAGTCCAA-TATGAGTA_S21	Ricaurte, Colombia	Female
JB_LM13-lutzomyia_male_25_021921_CGTACTAG-CTATTAAG_S60	Ricaurte, Colombia	Male
LU_LONG_F_P17_CAAGCTAG-ACATAGCG_S24	Ricaurte, Colombia	Female
LU_LONG_F_139_ACAGGCGC-CTCTGCCT_S6	Ricaurte, Colombia	Female
LU_LONG_F_P15_ATCCACTG-AGGTGCGT_S22	Ricaurte, Colombia	Female
JB_LF26-lutzomyia_female_93_021921_CTCTCTAC-TTATGCGA_S18	Ricaurte, Colombia	Female
JB_LF15-lutzomyia_female_82_021921_TAGGCATG-CTATTAAG_S6	Ricaurte, Colombia	Female
SRR3109093	Jacobina, Brazil	Female
SRR3109094	Jacobina, Brazil	Female
SRR3109095	Jacobina, Brazil	Female
SRR3109096	Jacobina, Brazil	Female
SRR3109097	Jacobina, Brazil	Female
SRR3109098	Jacobina, Brazil	Female
SRR3109099	Jacobina, Brazil	Female
SRR3109100	Jacobina, Brazil	Female
SRR3109101	Jacobina, Brazil	Female
SRR3109102	Jacobina, Brazil	Female
SRR3109103	Jacobina, Brazil	Female
SRR3109104	Jacobina, Brazil	Female
SRR3109105	Jacobina, Brazil	Female
SRR3109106	Jacobina, Brazil	Female
SRR3109107	Laphina, Brazil	Female
SRR3109108	Laphina, Brazil	Female

SRR3109109	Laphina, Brazil	Female
SRR3109110	Laphina, Brazil	Female
SRR3109111	Laphina, Brazil	Female
SRR3109112	Laphina, Brazil	Female
SRR3109113	Laphina, Brazil	Female
SRR3109114	Laphina, Brazil	Female
SRR3109115	Laphina, Brazil	Female
SRR3109116	Laphina, Brazil	Female
SRR3109117	Laphina, Brazil	Female
SRR3109118	Laphina, Brazil	Female
SRR3109119	Laphina, Brazil	Female
SRR3109120	Laphina, Brazil	Female
SRR3109121	Laphina, Brazil	Female
SRR3109122	Marajo, Brazil	Female
SRR3109123	Marajo, Brazil	Female
SRR3109124	Marajo, Brazil	Female
SRR3109125	Marajo, Brazil	Female
SRR3109126	Marajo, Brazil	Female
SRR3109127	Marajo, Brazil	Female
SRR3109128	Marajo, Brazil	Female
SRR3109129	Marajo, Brazil	Female
SRR3109130	Marajo, Brazil	Female
SRR3109131	Marajo, Brazil	Female
SRR3109132	Sobral 1S, Brazil	Female
SRR3109133	Sobral 1S, Brazil	Female
SRR3109134	Sobral 1S, Brazil	Female
SRR3109135	Sobral 1S, Brazil	Female

SRR3109136	Sobral 1S, Brazil	Female
SRR3109137	Sobral 1S, Brazil	Female
SRR3109138	Sobral 1S, Brazil	Female
SRR3109139	Sobral 1S, Brazil	Female
SRR3109140	Sobral 1S, Brazil	Female
SRR3109141	Sobral 1S, Brazil	Female
SRR3109142	Sobral 1S, Brazil	Female
SRR3109143	Sobral 1S, Brazil	Female
SRR3109144	Sobral 1S, Brazil	Female
SRR3109145	Sobral 1S, Brazil	Female
SRR3109146	Sobral 1S, Brazil	Female
SRR3109148	Sobral 2S, Brazil	Female
SRR3109149	Sobral 2S, Brazil	Female
SRR3109150	Sobral 2S, Brazil	Female
SRR3109151	Sobral 2S, Brazil	Female
SRR3109152	Sobral 2S, Brazil	Female
SRR3109153	Sobral 2S, Brazil	Female
SRR3109154	Sobral 2S, Brazil	Female
SRR3109155	Sobral 2S, Brazil	Female

SRR3109156	Sobral 2S, Brazil	Female
SRR3109157	Sobral 2S, Brazil	Female
SRR3109158	Sobral 2S, Brazil	Female
SRR3109159	Sobral 2S, Brazil	Female
SRR3109160	Sobral 2S, Brazil	Female
SRR3109161	Sobral 2S, Brazil	Female
SRR3109162	Sobral 2S, Brazil	Female
SRR3109163	Sobral 2S, Brazil	Female

Table S2. Significant GWAS hits. The table shows the name, location, association p-value, and known orthologs in other dipterans.

chrom	pos	p	gene_type	gene_id	gene_distance	Flybase_gene_name	gene_name
2	11287131	1.82E-08	Downstream	g4778	2049		
2	11287131	1.82E-08	Upstream	g4777	23544		
2	17800961	5.17E-09	Downstream	g5201	165		uncharacterized protein
2	17800961	5.17E-09	Upstream	g5200	35253		eye-specific diacylglycerol kinase-like
2	19878643	1.12E-09	Genic	g5391			
3	3650443	2.59E-09	Downstream	g6882	36835	CG17870	14-3-3 protein zeta
3	3650443	2.59E-09	Upstream	g6881	1646		
3	8753610	8.95E-08	Genic	g7272		CAA65152.1	mdg3 element ORF

3	8756972	4.43E-09	Genic	g7272		CAA65152.1	mdg3 element ORF
3	8757227	5.06E-08	Genic	g7272		CAA65152.1	mdg3 element ORF
3	14121068	2.91E-09	Downstream	g7711	14311		
3	14121068	2.91E-09	Upstream	g7710	14987		
3	14909442	1.22E-08	Downstream	g7789	4284		Orf2
3	14909442	1.22E-08	Upstream	g7788	6504	CG15715	zinc finger protein 706
3	14909452	4.62E-09	Downstream	g7789	4274		Orf2
3	14909452	4.62E-09	Upstream	g7788	6514	CG15715	zinc finger protein 706
3	15059348	4.94E-08	Downstream	g7799	3787		integrator complex subunit 12 #(low query coverage/confidence)
3	15059348	4.94E-08	Upstream	g7798	8033		uncharacterized protein K02A2.6-like
3	15061297	2.94E-08	Downstream	g7799	1838		integrator complex subunit 12 #(low query coverage/confidence)
3	15061297	2.94E-08	Upstream	g7798	9982		uncharacterized protein K02A2.6-like
3	15062377	2.89E-09	Downstream	g7799	758		integrator complex subunit 12 #(low query coverage/confidence)
3	15062377	2.89E-09	Upstream	g7798	11062		uncharacterized protein K02A2.6-like
3	27024829	7.38E-09	Downstream	g8767	7639	AAD37797	pleiotrophin isoform
3	27024829	7.38E-09	Upstream	g8766	15256		
3	27024841	3.28E-08	Downstream	g8767	7627	AAD37797	pleiotrophin isoform

3	27024841	3.28 E-08	Upstream	g8766	15268		
3	27024846	4.65 E-08	Downstream	g8767	7622	AAD37797	pleiotrophin isoform
3	27024846	4.65 E-08	Upstream	g8766	15273		
3	27024848	4.27 E-08	Downstream	g8767	7620	AAD37797	pleiotrophin isoform
3	27024848	4.27 E-08	Upstream	g8766	15275		
4	89619	6.71 E-08	Downstream	g8996	39230		na
4	89619	6.71 E-08	Upstream	g8995	10970		LIM/homeobox protein Lhx2 #(low confidence)
4	477670	1.12 E-09	Genic	g9046		CG6829	uncharacterized protein
4	477673	1.12 E-09	Genic	g9046		CG6829	uncharacterized protein
4	477798	1.03 E-08	Genic	g9046		CG6829	uncharacterized protein
4	477808	3.89 E-09	Genic	g9046		CG6829	uncharacterized protein
4	4318823	1.76 E-09	Downstream	g9424	853	CG2095	exocyst complex component 4
4	4318823	1.76 E-09	Upstream	g9423	355	AAL13990.1	heat shock factor protein
4	16129633	1.33 E-09	Downstream	g10362	4748	CG8333	enhancer of split mgamma protein
4	16129633	1.33 E-09	Upstream	g10361	18627		
4	16129745	1.13 E-09	Downstream	g10362	4636	CG8333	enhancer of split mgamma protein
4	16129745	1.13 E-09	Upstream	g10361	18739		
4	16129961	7.69 E-08	Downstream	g10362	4420	CG8333	enhancer of split mgamma protein
4	16129961	7.69 E-08	Upstream	g10361	18955		

4	16130036	1.61E-09	Downstream	g10362	4345	CG8333	enhancer of split mgamma protein
4	16130036	1.61E-09	Upstream	g10361	19030		
4	16130047	1.61E-09	Downstream	g10362	4334	CG8333	enhancer of split mgamma protein
4	16130047	1.61E-09	Upstream	g10361	19041		
4	16131132	1.82E-08	Downstream	g10362	3249	CG8333	enhancer of split mgamma protein
4	16131132	1.82E-08	Upstream	g10361	20126		
4	18327554	9.57E-09	Downstream	g10493	670	CG6975	tuberin
4	18327554	9.57E-09	Upstream	g10491	349	CG11660	serine/threonine-protein kinase RIO1
4	20445947	8.85E-09	Downstream	g10649	17732		
4	20445947	8.85E-09	Upstream	g10648	5814		uncharacterized protein
4	20445956	6.66E-10	Downstream	g10649	17723		
4	20445956	6.66E-10	Upstream	g10648	5823		uncharacterized protein

Table S3. GO categories showing enrichment in the GWAS hits.

**Enrichment**	**FDR**	**nGenes Pathway**	**Genes Fold**	**Enrichment Pathways**	**GO ID**
1.9E-03	3	106	49.4	Positive reg. of nervous system development	GO:0051962
9.6E-04	4	215	32.5	Reg. of nervous system development	GO:0051960
9.6E-04	4	242	28.9	Positive reg. of developmental proc.	GO:0051094
5.4E-03	3	197	26.6	Positive reg. of multicellular organismal proc.	GO:0051240

1.2E-03	4	281	24.9	Reg. of multicellular organismal development	GO:2000026
7.6E-03	3	234	22.4	Reg. of cell population proliferation	GO:0042127
3.3E-03	4	439	15.9	Response to abiotic stimulus	GO:0009628
1.4E-03	5	691	12.6	Reg. of developmental proc.	GO:0050793
5.9E-03	4	572	12.2	Reg. of multicellular organismal proc.	GO:0051239
1.1E-03	6	1176	8.9	Nervous system development	GO:0007399
2.0E-03	6	1443	7.3	Cell development	GO:0048468
9.6E-04	7	1739	7	Positive reg. of cellular proc.	GO:0048522
2.7E-03	6	1539	6.8	System development	GO:0048731
9.6E-04	7	1892	6.5	Positive reg. of biological proc.	GO:0048518
4.2E-03	6	1782	5.9	Cell differentiation	GO:0030154
4.2E-03	6	1790	5.9	Cellular developmental proc.	GO:0048869
4.2E-03	7	2831	4.3	Anatomical structure development	GO:0048856
5.3E-03	7	3005	4.1	Developmental proc.	GO:0032502
3.3E-03	8	4133	3.4	Reg. of cellular proc.	GO:0050794

SUPPLEMENTARY FIGURES

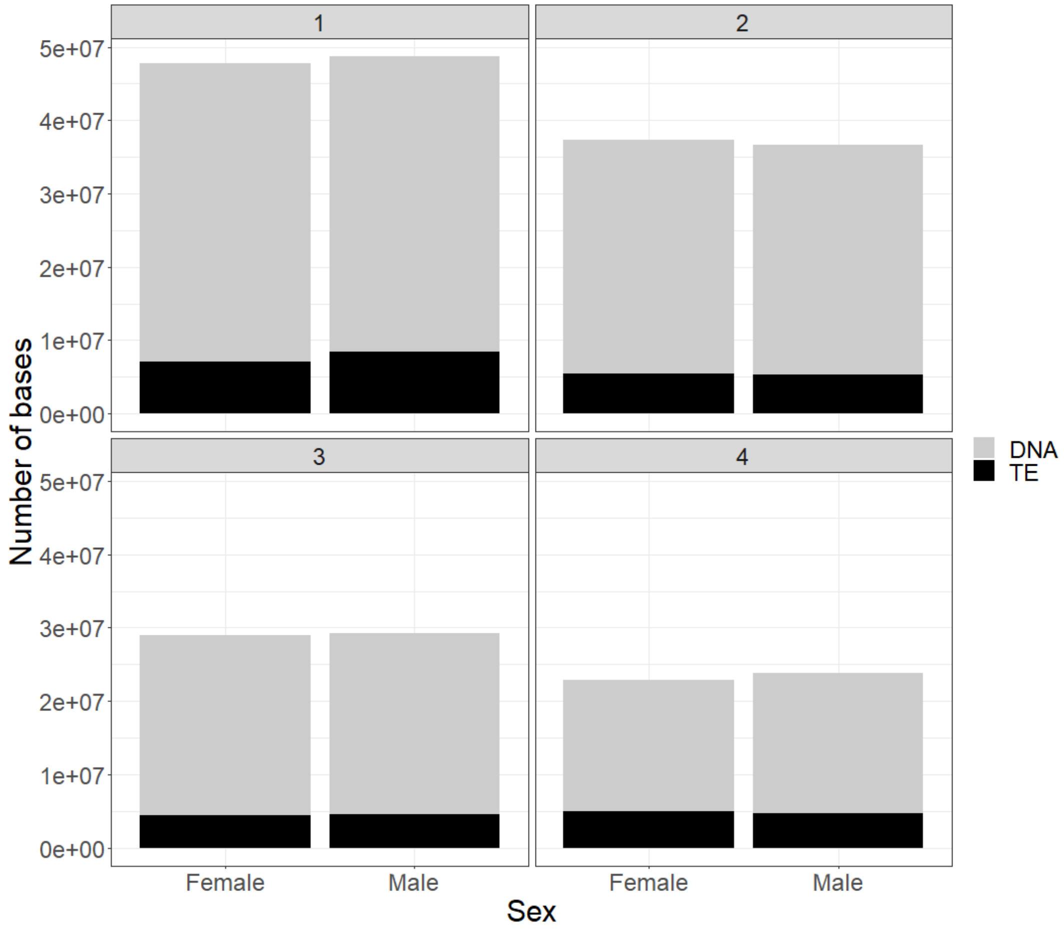

Figure S1. Overall number of bases classified as transposable elements (TE) and not classified as TE (DNA in figure legend). Facets represent the assembled chromosomes. Cumulative stacked bars represent total nucleotide bases per chromosome by sex.

Whole genome

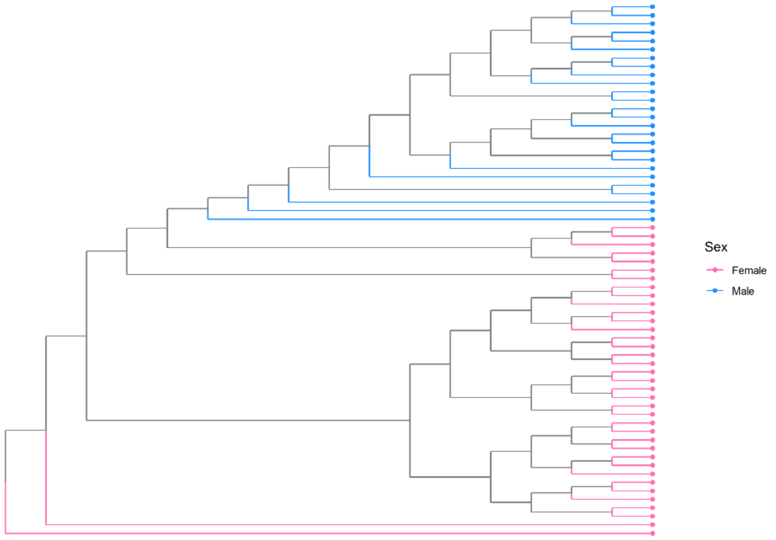

Chr1

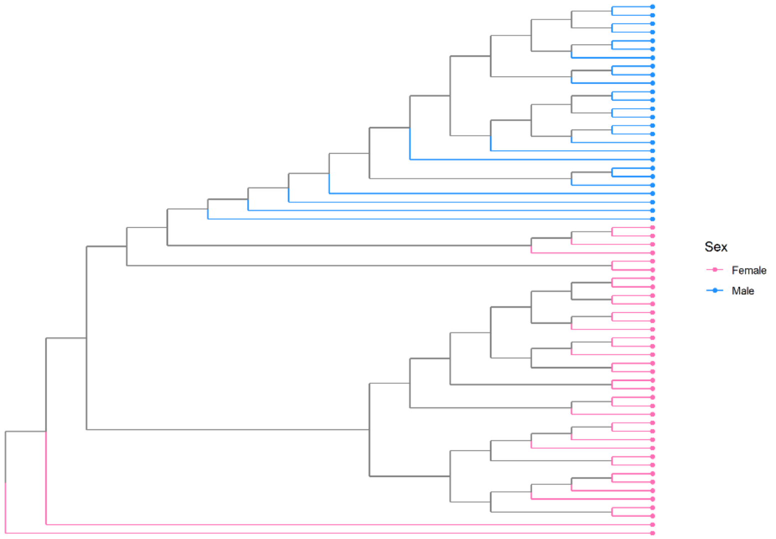

Autosomes

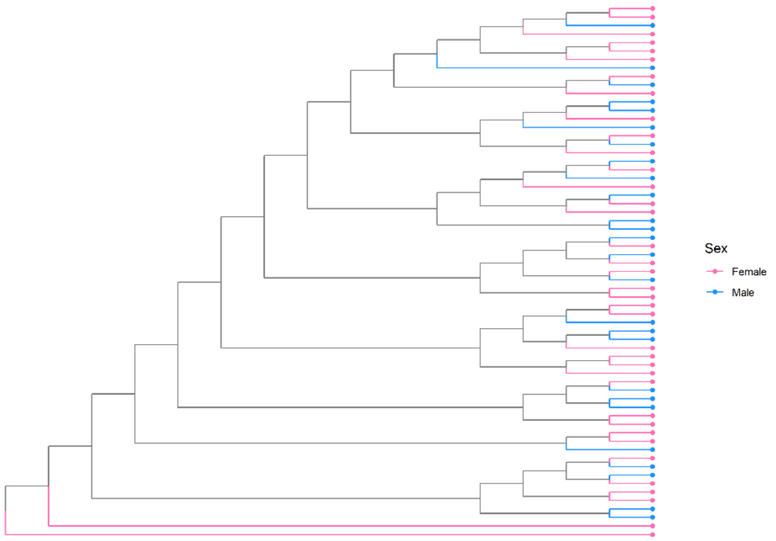

Figure S2. Phylogenetic trees of inter-relatedness between individuals across the whole genome, chromosome 1, and autosomes.

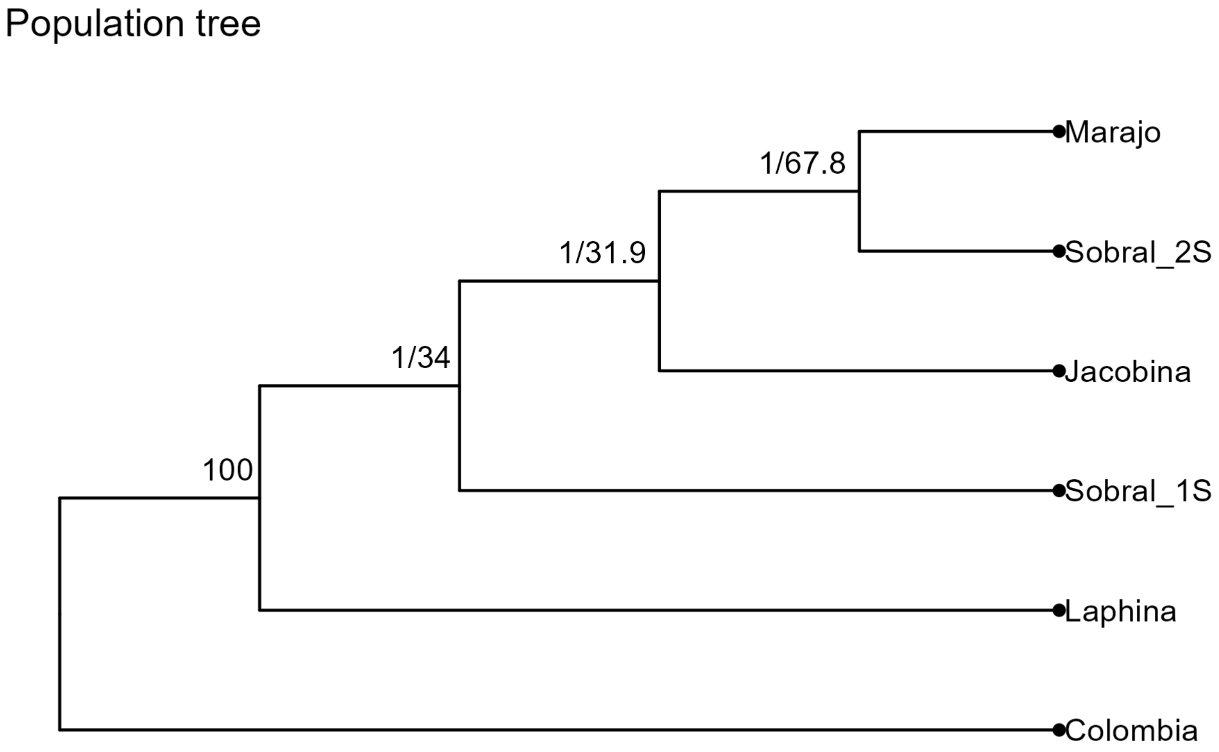

Figure S3. Consensus population tree from ASTRAL. The second values at the branches indicate gene concordance values from IQTREE2.

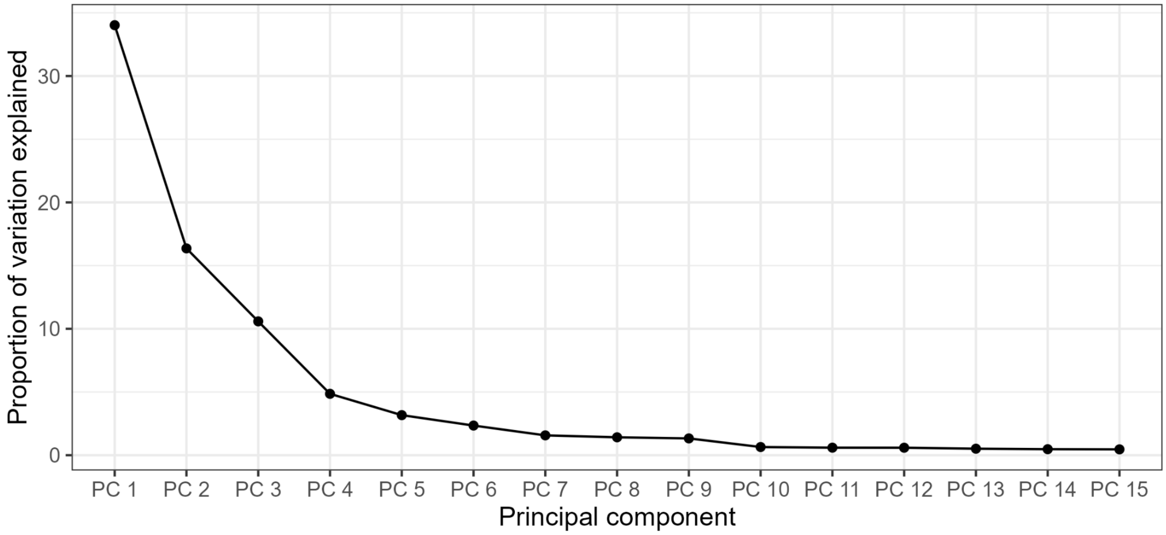

Figure S4. Scree plot of genomic variation in both Colombian and Brazilian sandflies explained. The proportion of variation explained is less than 3 after principal component 5.

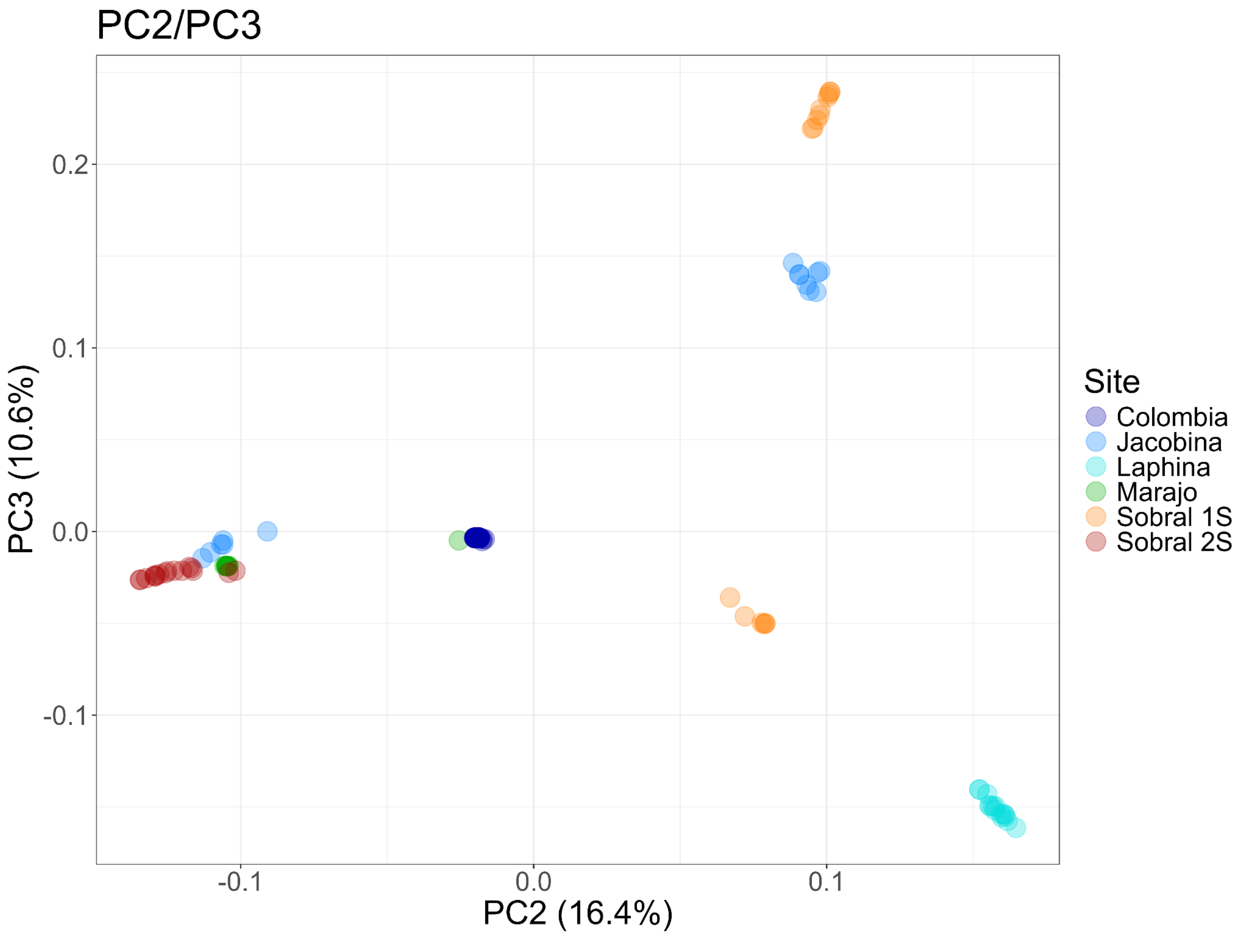

Figure S5. Principal components 2 and 3 from whole genome resequence data.

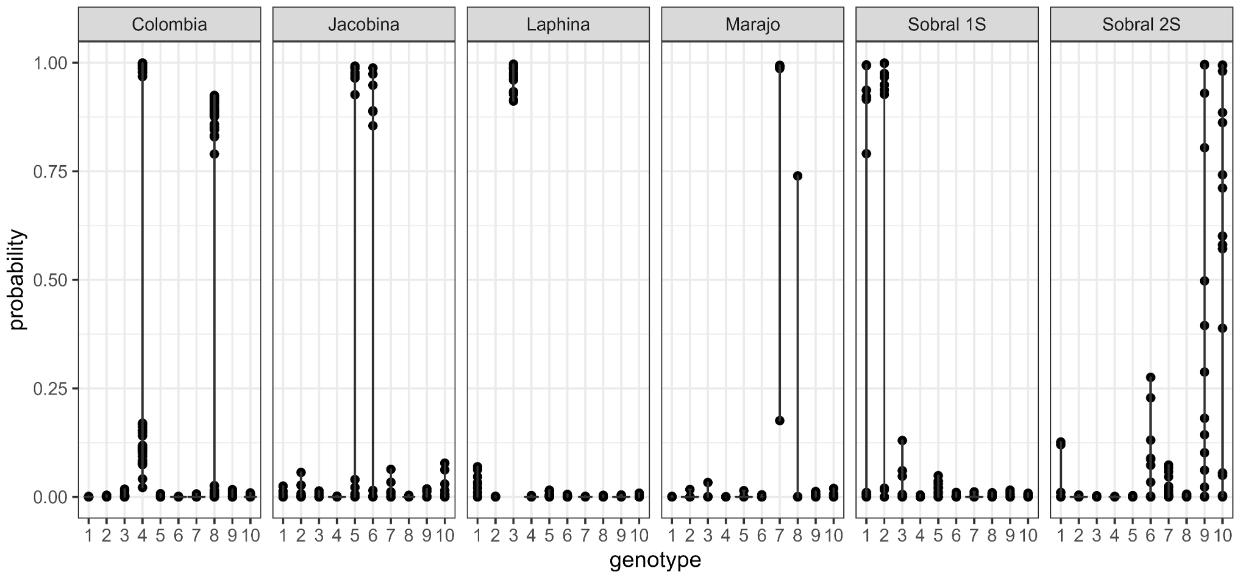

Figure S6. Admixture proportions by genotype for each individual sandfly.

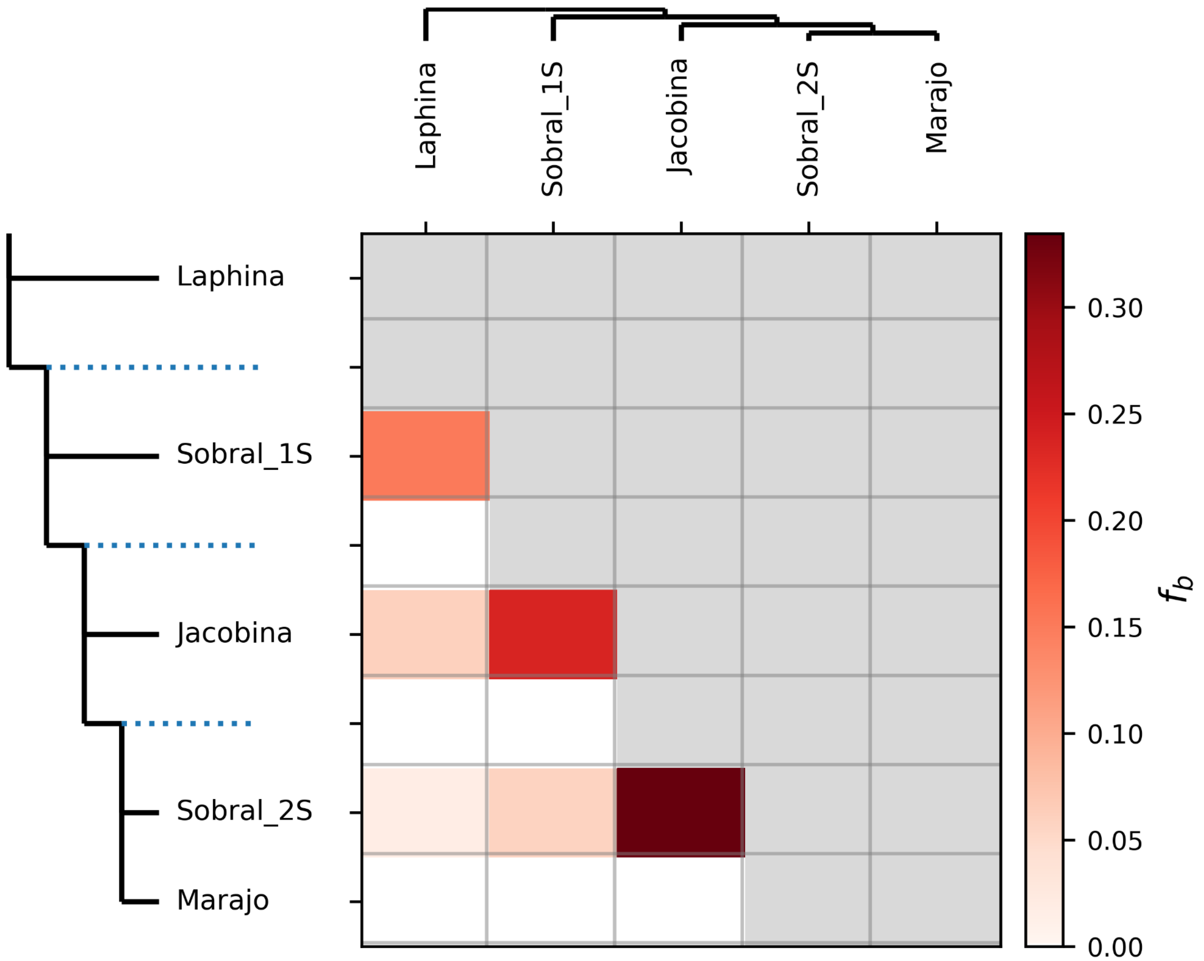

Figure S7. Fbranch statistics and gene flow inferred by Dsuite.

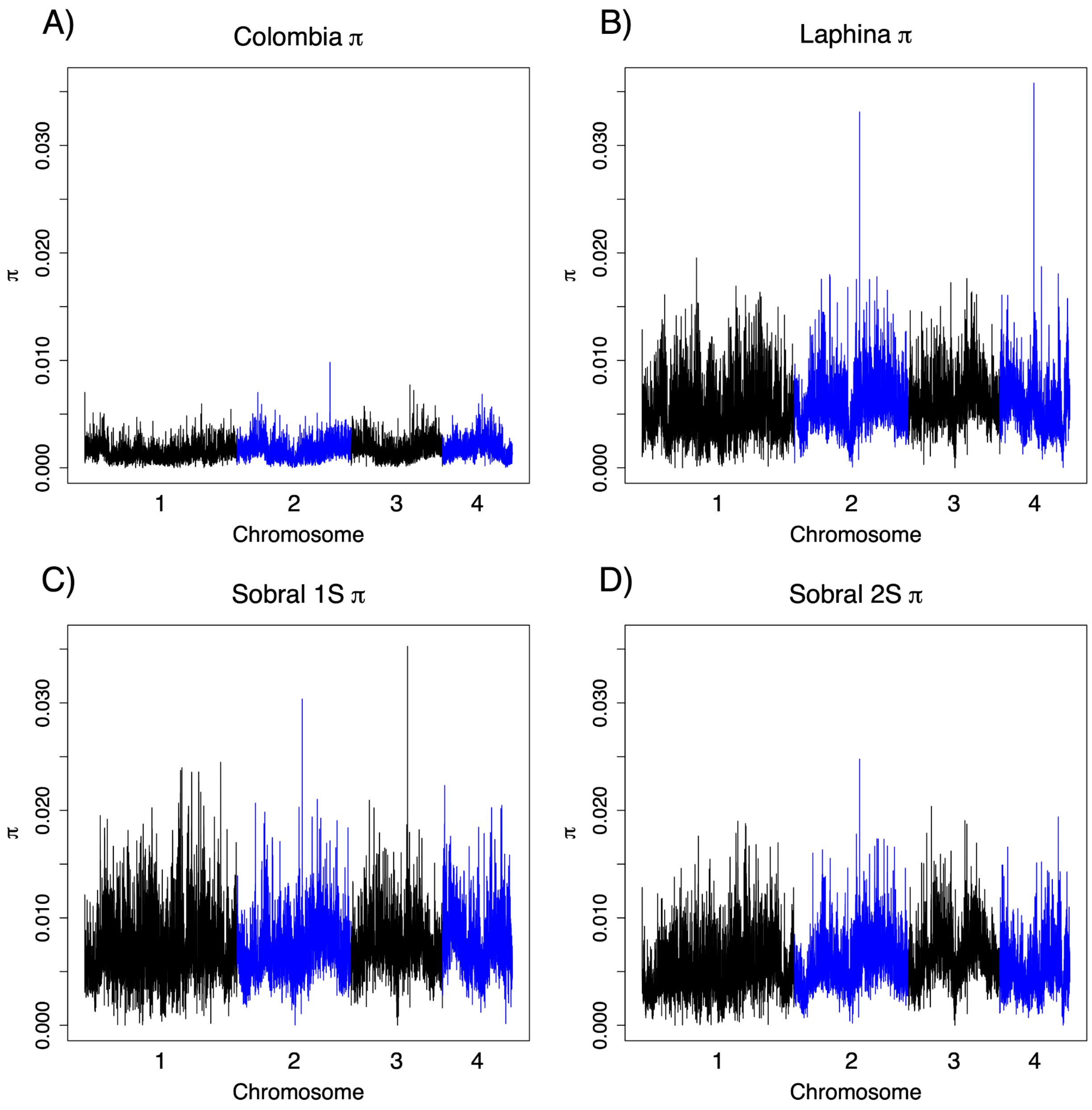

Figure S8. π in 10kb windows. **A.** π along the genome in Colombia. **B.** π along the genome in Laphina. **C.** π along the genome in Sobral 1S. **D.** π along the genome in Sobral 2S.

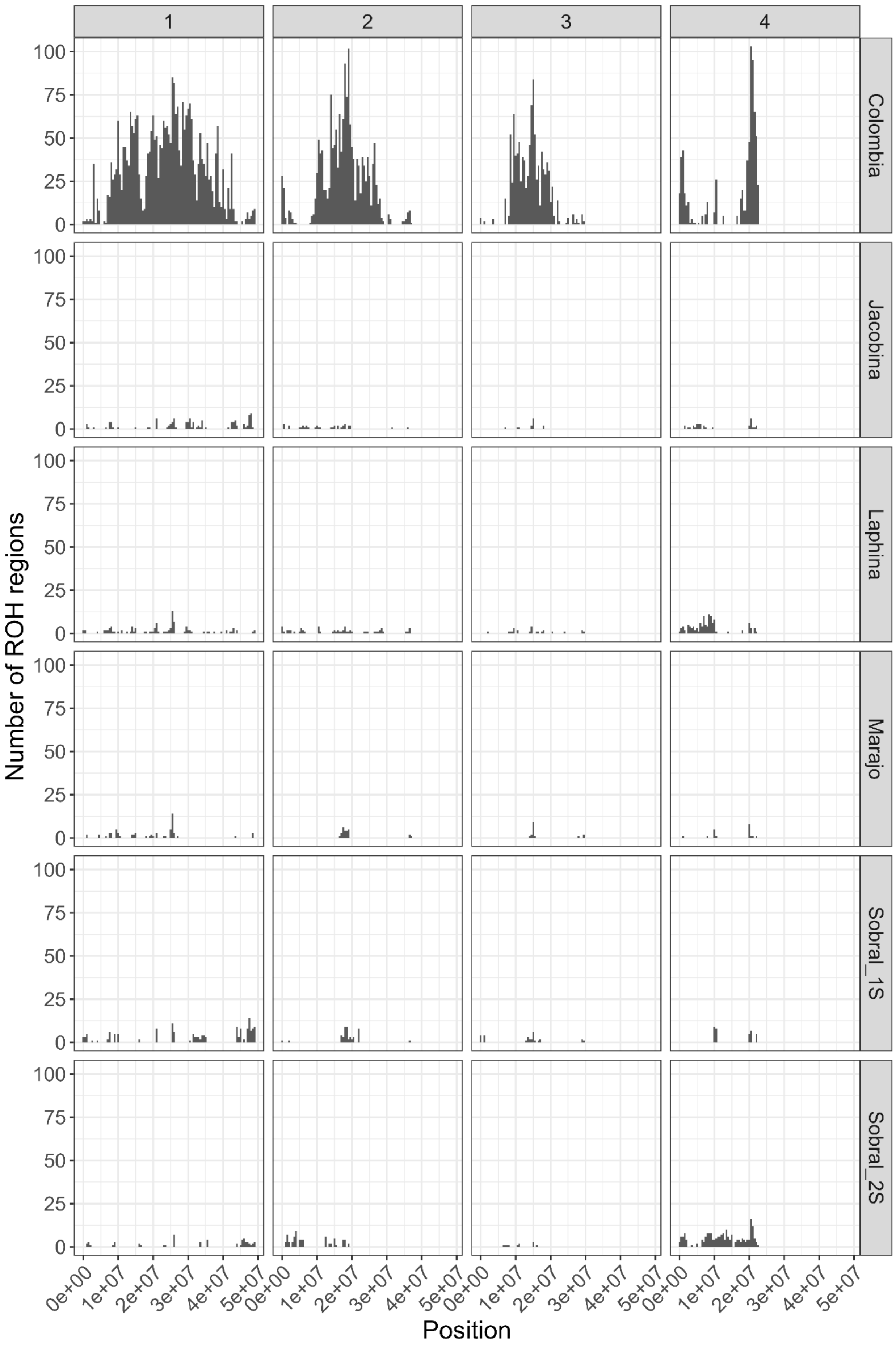
1
2
3
4
Colombia
Jacobina
Laphina
Marajo
Sobral_1S
Sobral_2S
Number of ROH regions
100
75
50
25
0
0e+00
1e+07
2e+07
3e+07
4e+07
5e+07
Position

Figure S9. Runs of Homozygosity in genomic regions from each population.

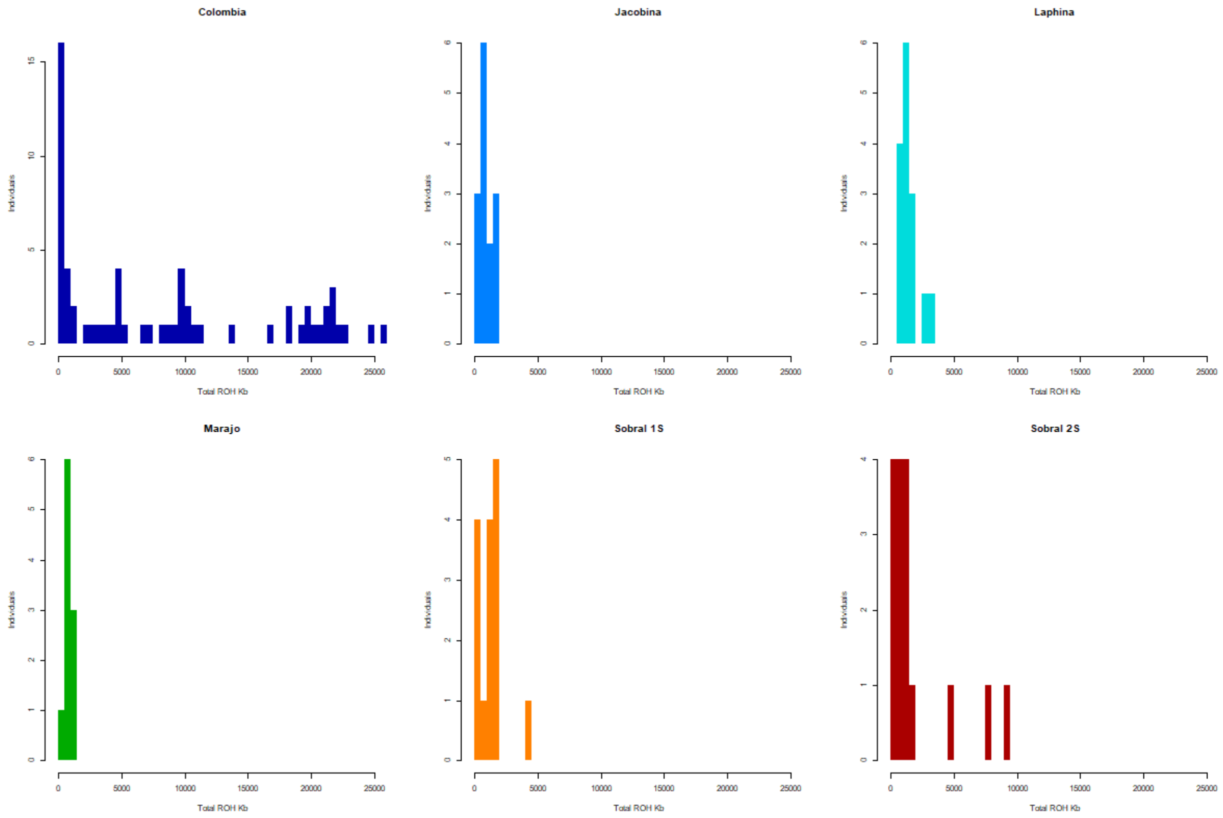

Figure S10. Histograms of ROH distributions by individual, clearly showing that the data from Colombia is not driven by a single individual.

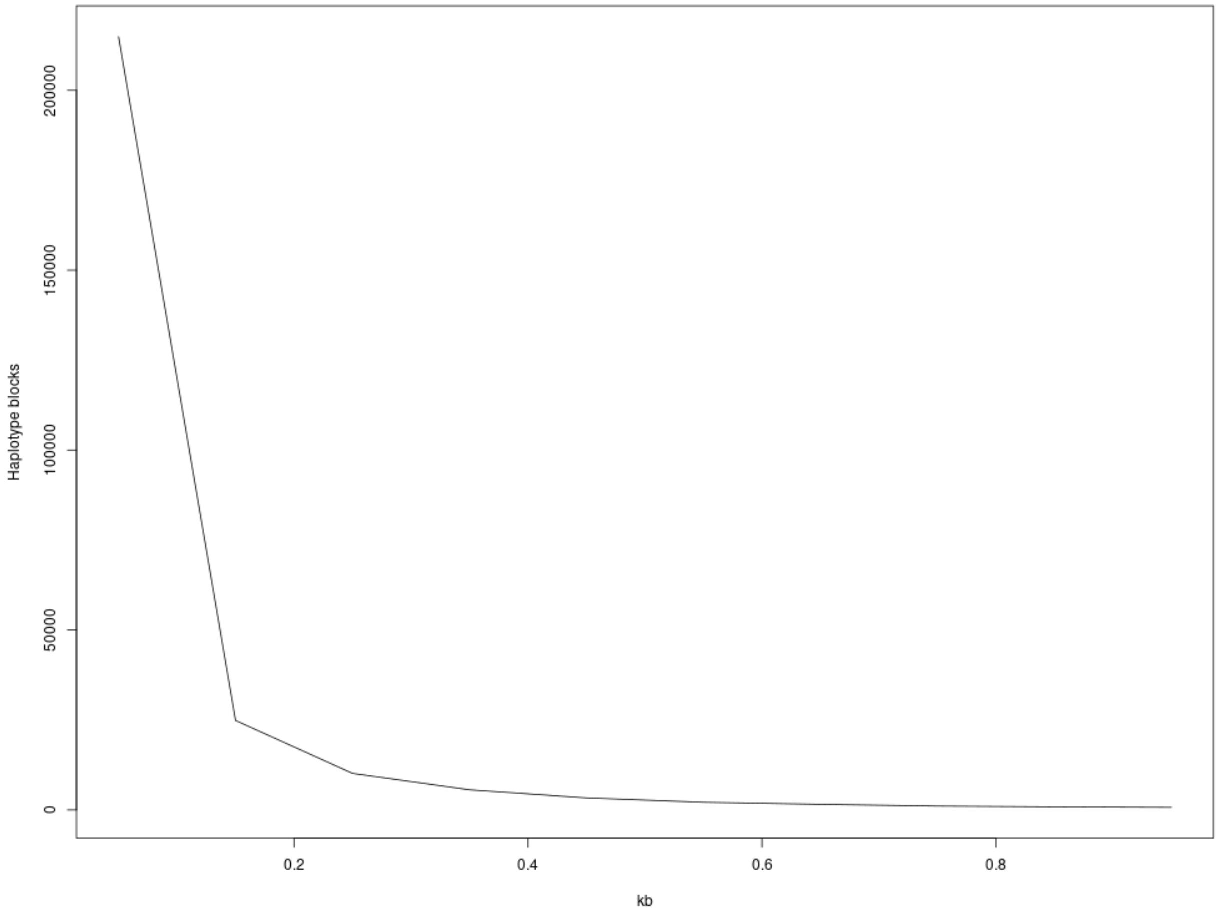

Figure S11. Linkage disequilibrium decays rapidly in *Lutzomyia longipalpis* with most haplotype blocks < 200bp.

DNA Extraction Protocol

Materials Needed:

- Zymo Genomic DNA Clean & Concentrator Kit
- Homogenization buffer (HB)
- 30mM Tris HCL (pH 8.0)
- 10mM EDTA
- 0:1M NaCl
- 0.5% Triton X-100
- SDS 10%
- RNase A
- Proteinase K

Note: This protocol is adapted from Dr. Jeremy Wang's high molecular weight extraction protocol for nanopore sequencing. Be sure that all solutions from the Zymo kit have the appropriate volume of 100% ethanol added.

1. Add 500 µL of HB to a 1:5mL tube with 20-40 frozen flies and then homogenize the flies using a clean pestle until you can no longer recognize any fly parts.
2. Add 125 µL of SDS and 10 µL of Proteinase K then vortex for 15 seconds.
3. Incubate at 55C for 1 hour (inverting every 10 minutes) and after 30 minutes add 10 µL of RNase A.
4. 4. Add twice the volume of ChIP DNA Binding Buffer to each volume of DNA sample and mix thoroughly.
5. Pipette the mixture into a Zymo-Spin Column in a collection tube, centrifuge at 16,000 x g for 30 seconds, and discard the flow-through.
6. Add 200 µL of DNA Wash Buffer to the Zymo-Spin Column, centrifuge at 16,000 x g for 1 minute, and discard the flow-through.
7. Repeat step 6.
8. Transfer the Zymo-Spin Column to a 1:5mL tube and to elute the DNA add 32 µL of DNA Elution Buffer directly onto the column matrix and centrifuge at 16,000 x g for 30 seconds.
9. Store the eluted DNA in the cold room until quantification.
